# New Whitening Constituents from Taiwan-Native *Pyracantha koidzumii*: Structures and Tyrosinase Inhibitory Analysis in Human Epidermal Melanocytes

**DOI:** 10.3390/ijms161226115

**Published:** 2015-12-02

**Authors:** Rong-Dih Lin, Mei-Chuan Chen, Yan-Ling Liu, Yi-Tzu Lin, Mei-Kuang Lu, Feng-Lin Hsu, Mei-Hsien Lee

**Affiliations:** 1 Department of Internal Medicine, Heping Branch, Taipei City Hospital, Taipei 100, Taiwan; lrd678@gmail.com; 2 Graduate Institute of Pharmacognosy, College of Pharmacy, Taipei Medical University, Taipei 110, Taiwan; mcchen1250@tmu.edu.tw (M.-C.C.); yann0119@hotmail.com (Y.-L.L.); acnnkiey1026@gmail.com (Y.-T.L.); hsu0320@tmu.edu.tw (F.-L.H.); 3 Ph.D. Program for the Clinical Drug Discovery from Botanical Herbs, College of Pharmacy, Taipei Medical University, Taipei 110, Taiwan; 4 National Research Institute of Chinese Medicine, Ministry of Health and Welfare, Taipei 112, Taiwan; mklu@nricm.edu.tw; 5 Center for Reproductive Medicine & Sciences, Taipei Medical University Hospital, Taipei 110, Taiwan

**Keywords:** *Pyracantha koidzumii*, 3,4-dihydroxy-5-methoxybiphenyl-2′-*O*-β-d-glucopyranoside, human epidermal melanocytes, tyrosinase, tyrosinase-related proteins, Paired box 3, microphthalmia-associated transcription factor

## Abstract

Nontoxic natural products useful in skin care cosmetics are of considerable interest. Tyrosinase is a rate-limiting enzyme for which its inhibitor is useful in developing whitening cosmetics. *Pyracantha koidzumii* (Hayata) Rehder is an endemic species in Taiwan that exhibits tyrosinase-inhibitory activity. To find new active natural compounds from *P. koidzumii*, we performed bioguided isolation and studied the related activity in human epidermal melanocytes. In total, 13 compounds were identified from *P. koidzumii* in the present study, including two new compounds, 3,6-dihydroxy-2,4-dimethoxy-dibenzofuran (**9**) and 3,4-dihydroxy-5-methoxybiphenyl-2ʹ-*O*-β-d-glucopyranoside (**13**), as well as 11 known compounds. The new compound **13** exhibited maximum potency in inhibiting cellular tyrosinase activity, the protein expression of cellular tyrosinase and tyrosinase-related protein-2, as well as the mRNA expression of Paired box 3 and microphthalmia-associated transcription factor in a concentration-dependent manner. In the enzyme kinetic assay, the new compound **13** acted as an uncompetitive mixed-type inhibitor against the substrate l-3,4-dihydroxyphenylalanine and had a *K_m_* value against this substrate of 0.262 mM, as calculated using the Lineweaver–Burk plots. Taken together, our findings show compound **13** exhibits tyrosinase inhibition in human melanocytes and compound **13** may be a potential candidate for use in cosmetics.

## 1. Introduction

Because of the concept of green consumers, the number of plant products has increased in the market and there is a greater demand in natural cosmetic products in most of the consumer markets recently. Skincare and health-related aspects of the problem are being increasingly focused on and the use of natural ingredients in cosmetics has become a current trend. Thus, the development of natural plant cosmetics has considerable potential.

Melanin is the black pigment in hair and skin and is synthesized from tyrosine by melanosomes [1]. Melanosomes are organelles in melanocytes at the dermis-epidermis junction. Because melanin formation is one of the main causes of skin darkening, controlling melanin synthesis is a crucial strategy in medical science and cosmetology [2]. The biosynthetic pathway of melanin involves the catalytic hydroxylation of tyrosine to l-3,4-dihydroxyphenylalanine (l-DOPA) by tyrosinase and the conversion of l-DOPA to dopaquinone. In the absence of thiol-containing compounds, dopaquinone converts initially to dopachrome and then to indole-5,6-quinone or indole-5,6-quinone-2-carboxylic acid. Tyrosinase-related protein-1 (TRP1; 5,6-dihydroxyindole-2-carboxylic acid (DHICA) oxidase; EC 1.14.18.) and tyrosinase-related protein-2 (TRP2/dopachrome tautomerase (DCT); EC 5.3.3.12) are involved in producing unstable quinones during the melanin polymerization process. Three major accessory enzymes of the tyrosinase family are involved in melanin biosynthesis [3,4].

Tyrosinase (EC 1.14.18.1) is a rate-limiting enzyme that is widely distributed in nature and is useful in developing whitening cosmetics [5,6]. Several studies have investigated the use of tyrosinase inhibitors, such as hydroquinone and its derivatives kojic acid, catechols, mercaptoamines, and alpha hydroxy acids, in cosmetic or pharmaceutical compositions for regulating skin pigmentation [7]. Tyrosinase is the most critical enzyme for pigment synthesis, and its levels show a marked response to UV radiation [8]. Thus, the development of agents that can modulate the enzymatic activity of tyrosinase will have considerable value in controlling the melanin contents in the skin [7]. Previous studies have demonstrated that tyrosinase is transcriptionally regulated by the microphthalmia-associated transcription factor (MITF), which leads to the synthesis of tyrosinase-related proteins [9]. Moreover, MITF is the key transcriptional regulator of multiple enzymes involved in melanogenesis [10].

Nontoxic natural products useful in formulating cosmetics and pharmaceuticals are of considerable interest. Plants are the main sources of natural cosmetics. Natural plant extracts, such as those from leaves, stems, cortices, petals, or fruits, can be used to protect human skin, in a similar role as that of nutrition and cosmetics [11].

*Pyracantha koidzumii* (Hayata) Rehder is a plant species of the family Rosaceae and is endemic to Taiwan. According to a few previous studies, the components isolated from *P. staudtii* may play a role in some of the traditional medicine remedies for threatened abortion and dysmenorrhea [12]. *P. crenulata* has an antiinflammatory effect [13]. Acylphloroglucinol and biphenyl glycosides were isolated from *P. fortuneana* [14,15]. Components such as carotenoids, flavonoids, glycosides, and sterol derivatives have been isolated from *Pyracantha* [13,14,15,16,17,18]. In particular, biphenyl glycosides were isolated from *Pyracantha* plants showing tyrosinase-inhibitory activity [15,17].

In a previous study, we found that an extract of *P. koidzumii* has low cytotoxic and higher cellular tyrosinase-inhibitory activity [19]. However, none of the active compounds from *P. koidzumii* investigated by the aforementioned studies demonstrates high tyrosinase-inhibitory activity. In the present study, the active compounds of *P. koidzumii* were isolated and tested for cellular anti-tyrosinase activity, and its effects on the expression of tyrosinase-related proteins, the related mRNA expression, and kinetic analysis in human epidermal melanocytes (HEMn) was studied.

## 2. Results and Discussion

**Figure 1:**
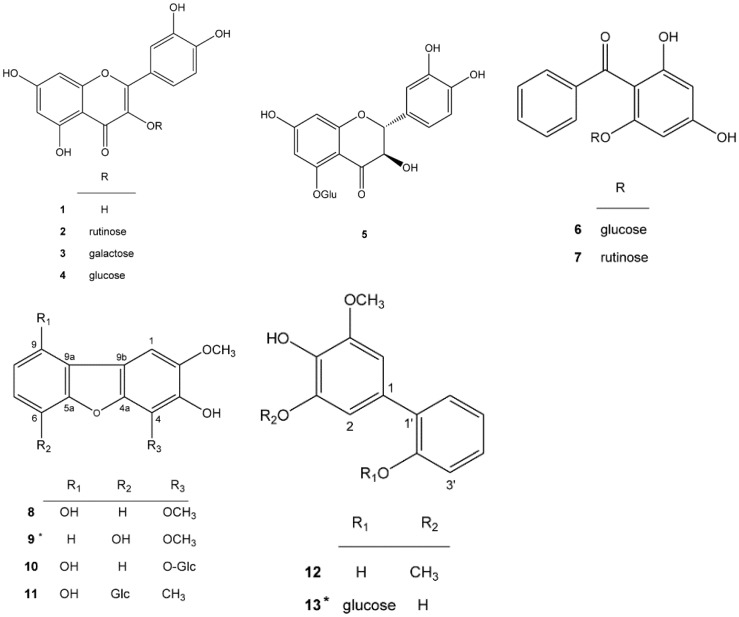
Structures of compounds isolated from *P. koidzumii*. (* new compound).

In our preliminary evaluation, the 95% ethanol fruit extract of *P. koidzumii* exhibited tyrosinase-inhibitory activity in HEMn cells [19]. In the present study, phytochemical investigations of *P. koidzumii* were conducted. Using a bioguided assay, we separately subjected the EtOAc and *n*-BuOH extracts to Diaion HP-20, Sephadex LH-20, MCI CHP-20P column chromatography, and semi-HPLC purification. Structure elucidation was achieved by comparing ^1^H- and ^13^C-NMR spectral data with literature data. Thirteen compounds, **1**–**13**, including two new compounds, **9** and **13**, were isolated from the active fractions. The structures of compounds **1**–**13** included five flavonoids (quercetin (**1**) [20], rutin (**2**) [21], hyperoside (**3**) [22], isoquercitrin (**4**) [22], and helicioside B (**5**) [23]), two diphenyl ketone glycosides (garcimangosone D (**6**) [24] and pyrafortunoside B (**7**) [14]), biphenyl and dibenzolfuran derivatives (9-hydroxyeriobofuran (**8**) [25], fortuneanoside L (**10**) [14], 2,4-dimethoxy-3,6,9-trihydroxy-dibenzofuranyl-6-*O*-β-d-glucopyranoside (**11**) [26], and 2-hydroxyaucuparin (**12**) [27]), and the two new compounds, **9** and **13** (Figure 1).

### 2.1. New Compounds **9** and **13** Isolated from Pyracantha koidzumii

Compound **9** was obtained as a pale yellow powder. The molecular formula was established to be C_14_H_12_O_5_ based on high-resolution electrospray ionization mass spectroscopy (HR-ESI-MS) (*m*/*z* 260.0687 [M]^+^, calculated for C_14_H_12_O_5_ 260.0679). The ^1^H-NMR spectrum of compound **9** showed typical signals of a 1,2,3-trisubstituted benzene ring (δ6.82 (1H, dd, *J* = 7.7, 1.0 Hz), δ7.08 (1H, t, *J* = 7.7 Hz), and δ7.33 (1H, dd, *J* = 7.7, 1.0 Hz)), a singlet signal (δ7.21, 1H) arising from a pentasubstituted benzene ring, and two singlet signals caused by *O*-methyl groups (δ3.94 (3H, s) and δ4.15 (3H, s)). The heteronuclear multiple bond coherence (HMBC) and heteronuclear multiple quantum coherence (HMQC) enabled assigning proton and carbon signals, δ6.82 (H-7) to δ146.0 (C-5a)/δ111.5 (C-8), δ7.08 (H-8) to δ144.1 (C-6)/δ127.9 (C-9a), and δ7.33 (H-9) to δ113.5 (C-7)/δ127.9 (C-9a). The *O*-linked aromatic quaternary carbon signals at δ144.1 and δ146.0 were correlated with H-8 and H7/H9, respectively; thus, they were assigned as C-6 and C-5a, respectively. The *O*-linked aromatic quaternary carbon signals at δ140.2 and δ144.6 were assigned as C-3 and C-4a, respectively, according to their HMBC correlations with H-1 (δ7.21, s). The chemical shifts of the *m*-substituted aromatic quaternary carbons C-3 and C-4a were upfield, indicating an *O*-substituted aromatic carbon at C-4; therefore, the remaining *O*-linked quaternary carbon signal at δ134.7 was assigned as C-4. The methoxy group at δ4.15 was located at C-4 according to the HMBC correlation, and the other methoxy group at δ3.94 was located at C-2 (the remnant *O*-linked quaternary carbon signal at δ147.4). Both aromatic quaternary carbon signals at δ127.9 and δ117.4 correlated with H-1/H-8 and H-9, suggesting that they were attributed to C-9a and C-9b, which were connected by two aromatic rings. Therefore, compound **9** was determined to be 3,6-dihydroxy-2,4-dimethoxy-dibenzofuran.

Compound **13** was obtained as a pale yellow powder. The molecular formula was established to be C_19_H_22_O_9_ based on HR-ESI-MS (*m*/*z* 394.1264, calculated value for C_19_H_22_O_9_ 394.1280). The ^1^H-NMR spectrum of compound **13** showed typical signals of a 1,2-bisubstituted benzene ring (δ7.02 (1H, m), δ7.22 (1H, dd, *J* = 7.6, 1.8 Hz), δ7.23 (1H, m), and δ7.27 (1H, dd, *J* = 8.4, 1.4 Hz)), metacouple protons (δ6.67 (1H, d, *J* = 1.8 Hz) and δ6.80 (1H, d, *J* = 1.8 Hz)) arising from a 1,3,4,5-tetrasubstituted benzene ring, and one singlet signal because of *O*-methyl groups (δ3.86, 3H). The aromatic protons at δ7.22, δ7.23, δ7.02, and δ7.27 were assigned as H-3′, H-4′, H-5′, and H-6′ based on the splitting pattern and COSY correlations. The HMBC and HMQC correlations of δ7.22 (H-3′) to δ133.0 (C-1′)/δ123.4 (C-5′), δ7.23 (H-4′) to δ155.4 (C-2′)/δ123.4 (C-5′), δ7.02 (H-5′) to δ133.0 (C-1′)/δ116.4 (C-3′), and δ7.27 (H-6′) to δ155.4 (C-2′)/δ129.0 (C-4′) further confirmed their location. The *O*-linked aromatic quaternary carbon signals at δ155.4 were correlated with H-4′ and H-6′; thus, they were assigned as C-2′. The remaining signals at δ3.34, δ3.42, δ3.43, δ3.44, δ3.68/δ3.86, and δ5.03 respectively correlated with the carbon signals at δ71.3 (C-4′′), δ78.2 (C-3′′), δ75.0 (C-2′′), δ78.3 (C-5′′), δ62.5 (C-6′′), and δ101.8 (C-1′′) in the HMQC spectrum. The results suggested the presence of a glucose residue in the structure of compound **13**. The acid hydrolysis of compound **13** further confirmed the structural elucidation. The HMBC experiments of compound **13** showed correlations between the anomeric protons at δ5.03 (1H, d, *J* = 7.2 Hz, H-1′′) and δ155.4 (C-2′), indicating a linkage of the β-d-glucopyranoside moiety to C-2′. In addition to the HMBC connectivity between the proton resonances at δ6.67 (H-2)/δ6.80 (H-6) and the ^13^C resonances at δ146.0, δ134.6/δ149.1, and δ134.6, the other ^1^H and ^13^C aromatic resonances confirm the existence of the H-2 and H-6 positions. The HMBC connectivity between δ3.86 and δ149.1 (C-5) confirms the presence of one methoxyl proton (δ3.86) at the C-5 position of the ring. Other *O*-linked aromatic quaternary carbon signals at δ134.6 and δ149.1 were assigned as C-4 and C-5, respectively, according to their respective HMBC correlations with H-2 and H-6. In these two benzylic components, we found HMBC correlations between δ6.80 (H-6) and δ133.0 (C-1′), δ6.67 (H-2) and δ133.0 (C-1′), and δ7.27 (H-6′) and δ130.6 (C-1). Therefore, compound **13** was determined to be 3,4-dihydroxy-5-methoxybiphenyl-2′-*O*-β-d-glucopyranoside.

### 2.2. Cell Viability of Human Epidermal Melanocytes Treated with Compounds Isolated from Pyracantha koidzumii

**Figure 2:**
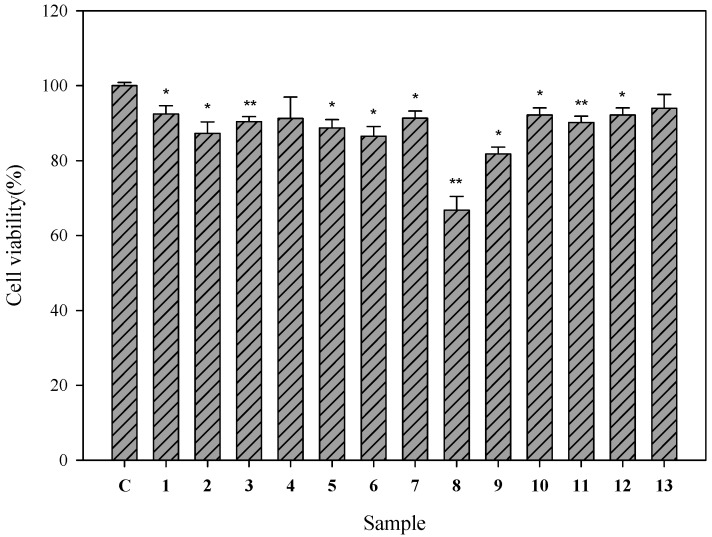
Cell viability of human epidermal melanocytes on treatment with compounds isolated from *P. koidzumii*. Cells (1 × 10^5^) were treated with 13 compounds (100 μM) for 24 h. After 24 h, the supernatant was removed and incubated with the WST-8 cell counting reagent for 4 h at 37 °C. The absorbance was measured at 450 nm by using a microplate reader. The cell viability (%) was calculated as follows: (OD_450_ of the sample/OD_450_ of control) × 100. Each determination was performed in triplicate and represented as mean ± SD. Differences in data were evaluated for statistical significance (* *p* < 0.05, ** *p* < 0.001) with the Student’s *t*-test. C: control.

To determine whether the test samples have cytotoxic effects on HEMn cells, their viability was initially evaluated using the WST-8 assay. Each of the isolated compounds from *P. koidzumii* was examined separately at 100 μM. All the compounds, except 9-hydroxyeriobofuran (**8**) (cell viability, 66.7%) preserved >80% of the cell viability (Figure 2). These 12 compounds exhibited less toxicity in the HEMn cells.

### 2.3. Cellular Tyrosinase-Inhibitory Activity and Melanin Content of the Isolated Compounds in Human Epidermal Melanocytes

**Figure 3:**
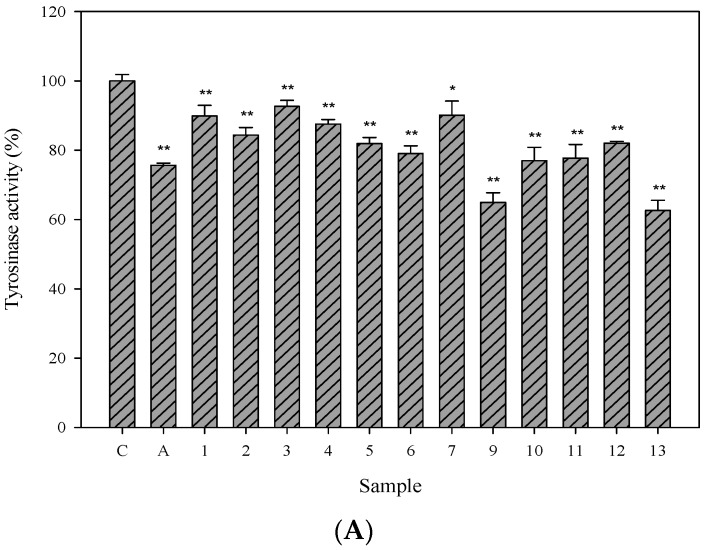

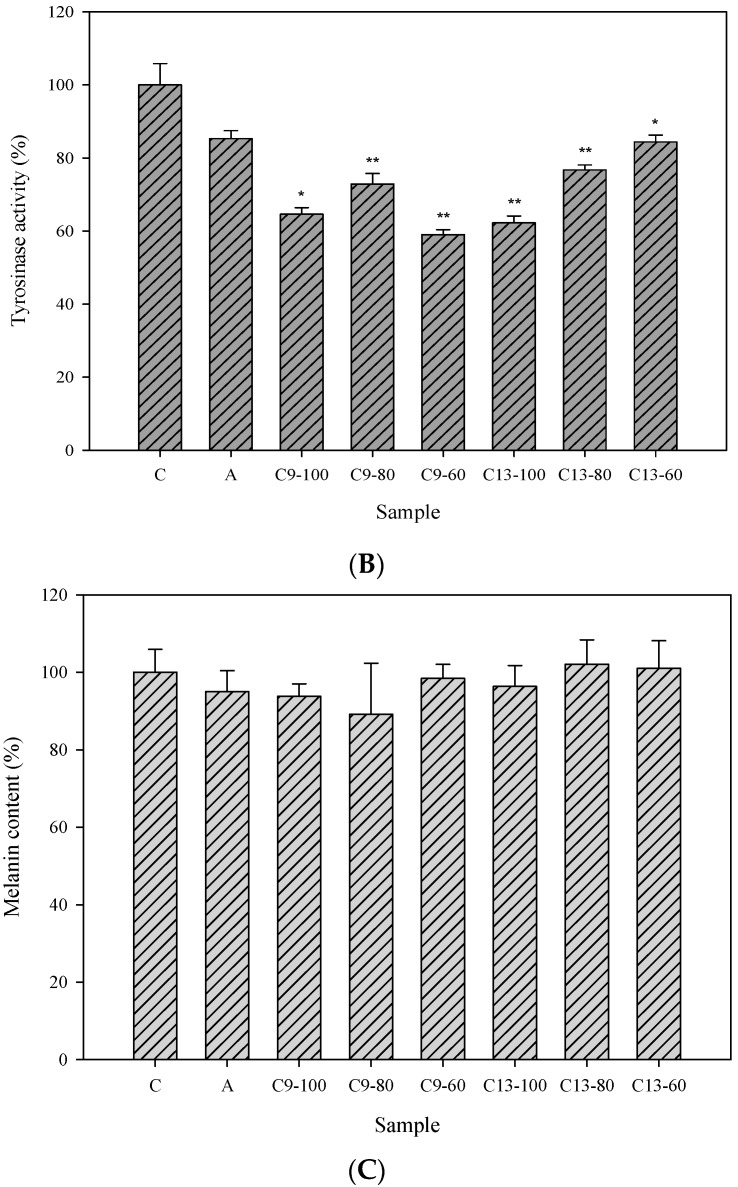
Cellular tyrosinase activities and melanin contents in human epidermal melanocytes. (**A**) tested compounds (100 μM); (**B**,**C**) 3,6-dihydroxy-2,4-dimethoxy-dibenzofuran (**9**) and 3,4-dihydroxy-5-methoxybiphenyl-2ʹ-*O*-β-d-glucopyranoside (**13**) (100, 80, and 60 μM). (**A**,**B**) Cells were treated with arbutin (IC_50_ 2.5 mM) and the tested compounds (100 μM) that yielded a cell viability higher than 80%. After 24 h, the cells were harvested. The lysates (with equal amounts of protein) were incubated with l-DOPA at a final concentration of 2 mM for 1 h at 37 °C. The tyrosinase activity (%) was calculated as follows: (OD_475_ of the sample/OD_475_ of control) × 100. Each determination was performed in triplicate and represented as mean ± SD; (**C**) Cell pellets were dissolved in 1 N NaOH at 37 °C overnight for measuring the melanin contents. The optical densities of the supernatants were measured at 450 nm. Differences in data were evaluated for statistical significance (* *p* < 0.05, ** *p* < 0.001) with the Student’s *t*-test. C: control, A: arbutin, C9: 3,6-dihydroxy-2,4-dimethoxy-dibenzofuran (**9**), C13: 3,4-dihydroxy-5-methoxybiphenyl-2′-*O*-β-d-glucopyranoside (**13**).

Tyrosinase is the rate-limiting enzyme in melanin synthesis and its inhibitor is used as a major ingredient in developing new whitening agents. Therefore, we further evaluated the cellular tyrosinase-inhibitory activity of isolated compounds exhibiting less toxicity in the HEMn cells. Arbutin (2.5 mM), the commercial whitening agent, was used as the positive control. Among the isolated compounds, compounds **9** and **13** exhibited potent cellular tyrosinase-inhibitory activity (Figure 3A). Compound **13** showed concentration-dependent cellular tyrosinase-inhibitory activity within a range of 60–100 μM (Figure 3B). The melanin contents of compounds **9** and **13** are shown in Figure 3C; there were no statistically significant differences between them.

### 2.4. Effects of 3,6-Dihydroxy-2,4-dimethoxy-dibenzofuran (**9**) and 3,4-Dihydroxy-5-methoxybiphenyl-2′-O-β-d-glucopyranoside (**13**) on the Expression of Tyrosinase-Related Proteins in Human Epidermal Melanocytes

**Figure 4:**
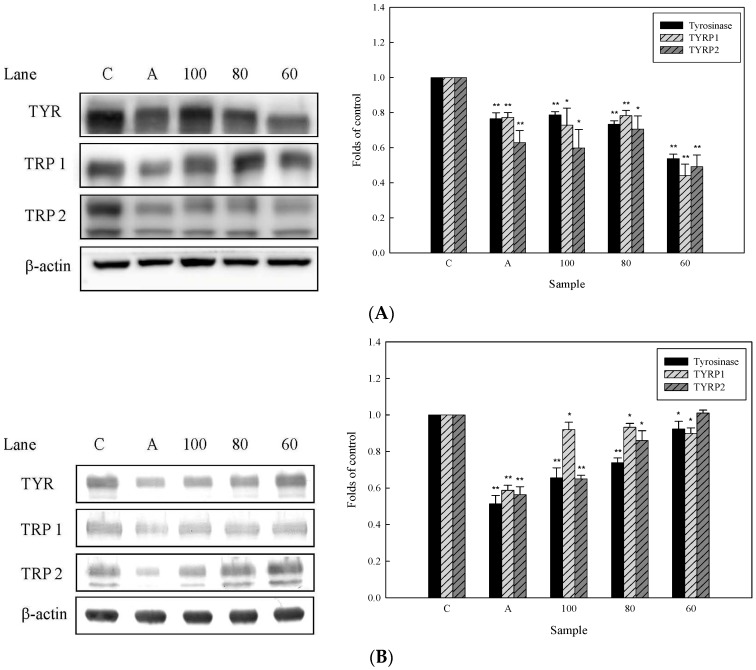
Western blot analysis of tyrosinase-related proteins in human epidermal melanocytes treated with (**A**) 3,6-dihydroxy-2,4-dimethoxy-dibenzofuran (**9**); and (**B**) 3,4-dihydroxy-5-methoxybiphenyl-2′-*O*-β-d-glucopyranoside (**13**). Cells (1 × 10^6^) were treated with different concentrations of compounds **9** and **13** for 24 h. Cells were then harvested, and the lysates (with equal amounts of protein) were electrophoresed using sodium dodecyl sulfate-10% polyacrylamide gels, followed by electroblotting and immunostaining with antibodies against tyrosinase, tyrosinase-related protein-1 (TRP1), tyrosinase-related protein-2 (TRP2), and β-actin. C: control, A: arbutin. Densitometry values (right) are presented as the mean ± S.D. of triplicate independent experiments. * *p* < 0.05, ** *p* < 0.001 as compared with control group.(100: 100 µM, 80: 80 µM, 60: 60 µM).

Because melanin is one of the heteropolymers produced inside melanosomes by the tyrosinase enzyme that acts on the tyrosinase precursors in melanocytes, we further studied the hypopigmentary effect of compounds **9** and **13**. Some metal ions played a cofactor role for the activity of tyrosinase enzyme and tyrosinase enzymes (tyrosinase, TRP1, and TRP2) were reported to affect melanin production [28]. TRP2 is reported to function as a dopachrome tautomerase downstream of tyrosinase in the melanogenic pathway and is related to the quantity and quality of melanin produced during melanin biosynthesis [29,30]. These proteins constitute a specific family of membrane proteins that are structurally related but have distinct enzymatic functions [31]. The effects of compounds **9** and **13** on these proteins after 24 h of treatment were evaluated by western blot analysis. HEMn cells were exposed to various concentrations of compounds **9** and **13** (60, 80, and 100 µM), and the reduction in activity on treatment with compounds **9** and **13** was compared with that on treatment with the control preparations by using the Quantity One 1-D Analysis Software. Based on the present study, compound **13** was found to decrease the levels of the pigment-related proteins tyrosinase and TRP2 in a concentration-dependent manner (Figure 4B), while compound **9** exhibited the most potent response at 60 µM in inhibition of tyrosinase, TRP1, and TRP2 expression (Figure 4A), suggesting the complex mode of action of compound **9** in regulating tyrosinase-related proteins expression relative to compound **13** examined.

### 2.5. Effects of 3,6-Dihydroxy-2,4-dimethoxy-dibenzofuran (**9**) and 3,4-Dihydroxy-5-methoxybiphenyl-2′-O-β-d-glucopyranoside (**13**) on the Expression of MITF and PAX3 mRNA in Human Epidermal Melanocytes

**Figure 5:**
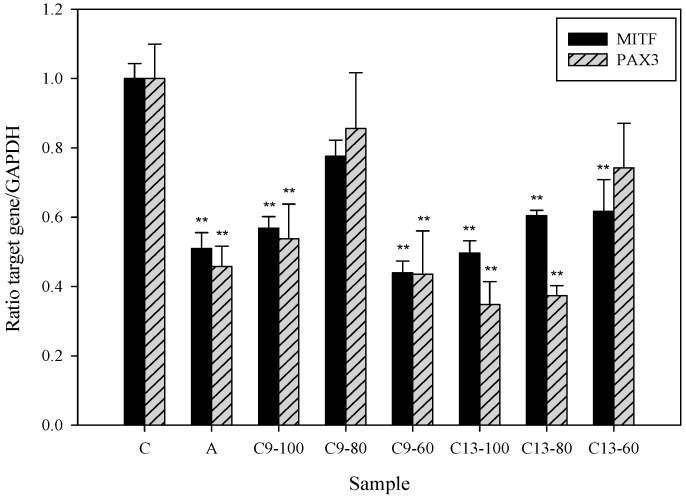
Real-time PCR analysis of microphthalmia-associated transcription factor (MITF) and Paired box 3 (PAX3) in human epidermal melanocytes treated with 3,6-dihydroxy-2,4-dimethoxy-dibenzofuran (**9**) and 3,4-dihydroxy-5-methoxybiphenyl-2′-*O*-β-d-glucopyranoside (**13**). Cells (1 × 10^6^) were treated with different concentrations (100, 80, and 60 µM) of compounds **9** and **13** for 24 h. Quantification of gene transcripts was performed using a LightCycler^®^ 480 TaqMan according to the manufacturer’s instructions. Findings were normalized to the expression of GAPDH mRNA. Measurements were conducted in triplicate, and mean expression values for test samples relative to mean expression values for negative controls are indicated. C: control, A: arbutin (2.5 mM), C9: 3,6-dihydroxy-2,4-dimethoxy-dibenzofuran (**9**), C13: 3,4-dihydroxy-5-methoxybiphenyl-2′-*O*-β-d-glucopyranoside (**13**). Differences in data were evaluated for statistical significance (** *p* < 0.001) with the Student’s *t*-test.

In addition to important roles of TRP1 and TRP2 for melanin synthesis, a previous report has indicated that transcription factor MITF has the ability to regulate expression levels of TRP1, TRP2, and tyrosinase by transactivating those genes [32]. MITF plays a major role in melanogenesis by regulating the extracellular signal-regulated kinase and AKT/protein kinase B signaling [33] and also transcriptionally regulates the expression of the tyrosinase-related proteins [34]. Our data showed that compound **13** dose-dependently inhibits MITF mRNA expression in HEMn cells (Figure 5). It is well-studied that transcription factor PAX3 (Paired box 3) can synergize with Sox10 to strongly activate MITF expression [35,36]. To investigate the effect of our compounds on PAX3, we further examined the expression level of PAX3 in compound **13**-treated HEMn cells. The dose-dependent suppressive effect of compound **13** on PAX3 mRNA expression was demonstrated in Figure 5, suggesting compound **13**-mediated MITF suppression may be through reduction of PAX3 mediated-transcriptional activity. Interestingly, treatment with a range of concentrations of compound **9** also revealed a biphasic effect on PAX3 and MITF mRNA expression levels, *i.e.*, 60 μM of compound **9** exhibits more potent inhibition activity than higher concentrations (80 and 100 μM) of compound **9**. In addition, evidence indicates compound **9** has slightly cytotoxicity induction in HEMn cells (Figure 2).

### 2.6. Tyrosinase Kinetic Analysis on 3,4-Dihydroxy-5-methoxybiphenyl-2ʹ-O-β-d-glucopyranoside (**13**) Treatment of Human Epidermal Melanocytes

**Figure 6:**
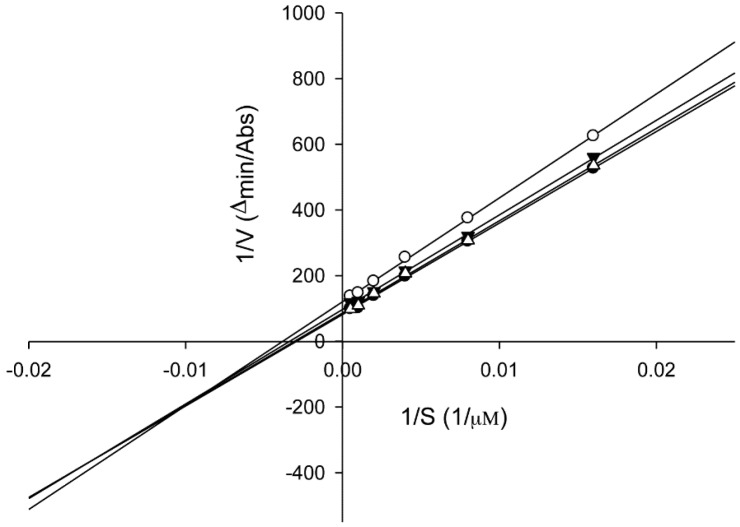
Lineweaver-Burk plots of cellular tyrosinase in the absence and presence of 3,4-dihydroxy-5-methoxybiphenyl-2′-*O*-β-d-glucopyranoside (**13**). Data were obtained as mean values of 1/*V*, the inverse of the increase in absorbance at 495 nm·min^−1^ (∆OD_495_ min^−1^). Cells (1 × 10^5^) were treated with different doses (60, 80, and 100 μM) of compound **13** for 24 h and harvested. Proteins (40 μg) were incubated with l-DOPA at a final concentration of 62.5, 125, 250, 500, 1000, and 2000 μM for 1 h at 37 °C. Three independent tests were conducted with different concentrations of l-DOPA as the substrate. The absence (●); and presence of compound **13** at 100 μM (○); 80 μΜ (▼); or 60 μM (∆).

To examine the mechanism of action, we performed an enzyme kinetic study on compound **13** by performing HEMn-based tyrosinase assays with various concentrations of the substrate l-DOPA (0.0625, 0.125, 0.25, 0.5, 1, and 2 mM). A Lineweaver–Burk plot of the data is shown in Figure 6; the *K*_m_ and *V*_max_ values were calculated to be 3.40 × 10^2^ μM and 1.22 × 10^−2^ μM·min^−1^, respectively, for no inhibition. On treatment with various concentrations of compound **13** (60, 80, and 100 μM), the *K*_m_ values were 3.26 × 10^2^, 2.96 × 10^2^, and 2.61 × 10^2^ μM, respectively and the *V*_max_ values were 1.16 × 10^−2^, 1.03 × 10^−2^, and 8.27 × 10^−3^ ∆A·min^−1^, respectively. Compound **13** acts as a mixed-type inhibitor against the substrate l-DOPA at 60, 80, and 100 μM concentrations. These results indicated that compound **13** could bind to the enzyme and the enzyme-substrate complex in a concentration-dependent manner [37]. For inhibiting tyrosinase, the compound may function through two alternative mechanisms, competitive and uncompetitive modes [38].

Melanogenesis is multi-directionally regulated via pathways activated by receptor-dependent and -independent mechanisms [38]. Evidence has shown that l-tyrosine and l-dopa act as positive regulators to play important roles in the melanogenic pathway through receptor-or non-receptor-mediated signaling [39]. Mechanisms of regulation of melanogenesis are involved in transcriptional regulation, including genes for tyrosinase and several melanogenesis-related proteins (MRPs) [38,40]. Importantly, regulation of gene mRNA expression level only contributes the initial steps; posttranslational modifications play the crucial roles of the final regulation of melanin synthesis [38,41]. In addition, various intracellular signal transduction mechanisms are also involved in melanogenesis regulation [38]. cAMP acts as a critical factor to activate protein kinase A (PKA) to promote phosphorylation of enzymes, ion channels, and various regulatory proteins [38,42]. However, it has been reported that cAMP can inhibit melanogenesis via PKA-independent p21Ras activation [43], indicating a complex regulatory mechanism of melanogenesis.

In this study, our results show that one of the active components—compound **13**—inhibits tyrosinase activity in human epidermal melanocytes without inducing cytotoxicity. It has been shown that MITF is the most important transcriptional regulator for driving the numerous signals involved in the expression of genes related to melanogenesis [44]. MITF activates essential regulators for melanin production, such as tyrosinase family genes TYR, TRP1, and TRP2 [45]. In addition, MITF also plays an important role in melanocyte development, proliferation, and survival by regulating bcl2, p21, and CDK2 [45]. Compound **13** also suppresses mRNA expression level of MITF and downstream TRP1, TRP2, and tyrosinase protein levels in HEMn cells. Further, downregulation of upstream transcription factor PAX3 has been observed in compound **13**-treated cells, suggesting PAX3 may play a role in compound **13**-triggered downregulation of TRP1, TRP2, MITF, and tyrosinase. However, it has been reported that PAX3 did not show appreciable expression in melanocyte of normal skin [46,47] and some studies indicated PAX3 is expressed in melanomas to support malignant cell survival [46,47,48]. In addition, Wnt signaling has been reported to induce transcriptional activation of MITF in melanocyte [49]. Further, signal transducer and activator of transcription 3 (STAT3) and cAMP response element binding protein (CREB) may influence the transcription activity of MITF [50], suggesting compound **13**-regulated signaling and effects can be further investigated. Our findings demonstrate that compound **13**, compared with compound **9**, exhibits dose-dependent inhibition of tyrosinase activity, TRP1, TRP2, MITF, and the critical transcription factor PAX3. However, compound **9** shows obvious inhibitory effects at lower concentration (60 μM) as shown in our results. It is possible the underlying chemical mechanism (e.g., solubility) plays the crucial role to determine the biphasic effect of compound **9**. Taken together, these findings indicate that compound **13** exhibits better tyrosinase inhibition than compound **9** to be further developed in the future.

## 3. Experimental Section

### 3.1. Reagents

Acrylamide, ammonium persulfate, aprotinin, bromophenol blue, dimethyl sulfoxide (DMSO), l-DOPA, 2-(2-methoxy-4-nitrophenyl)-3-(4-nitrophenyl)-5-(2,4-disulfophenyl)-2*H*-tetrazolium, monosodium salt (WST-8), ethylenediaminetetraacetic acid, formaldehyde, glycerol, leupeptin, 2-mercaptoethanol, polyacrylamide, and Triton X-100 were purchased from Sigma-Aldrich (St. Louis, MO, USA). The other chemicals and reagents used in the study were high-grade commercial products.

### 3.2. Collection, Extraction, and Isolation

Fruits of *P.* *koidzumii* were collected during November 2007 from the Highlands Experiment Farm, National Taiwan University, Nantou, Taiwan, and identified by Mr. Chi-Luan Wen, Seed Improvement and Propagation Station, Council of Agriculture, Taiwan. A voucher specimen number (M-119) was deposited in the Graduate Institute of Pharmacognosy, College of Pharmacy, Taipei Medical University.

The fruits were pressed and then extracted with 95% ethanol three times. The resulting ethanol solutions were combined and concentrated under reduced pressure to obtain a 95% ethanol raw extract. The raw extract was suspended in water and then extracted with *n*-hexane, ethyl acetate (EtOAc), and *n*-butanol (*n*-BuOH) in order. The EtOAc extract (25.88 g) was resuspended in water and isolated using a Diaion HP-20 column by a gradient elution of 100% water to 100% methanol. The resulting eluates were assayed on a thin-layer chromatography (TLC) (Merck KGaA, Darmstadt, Germany) plate (CH_2_Cl_2_/Methanol/Acetic acid = 7:1:0.1), and seven fractions (PK-1-1 to PK-1-7) were obtained. The fractions PK-1-3 (1.99 g), PK-1-4 (1.15 g), and PK-1-5 (15.42 g) were further isolated using a C_18_ column by a gradient elution of 100% water to 100% methanol, and the following fractions were obtained: PK-1-3-1 to PK-1-3-8, PK-1-4-1 to PK-1-4-8, and PK-1-5-1 to PK-1-5-10. PK-1-3-5 (0.25 g) was further purified using semipreparative HPLC (Hitachi, Tokyo, Japan) with a Biosil 5 ODS-W column (10 × 250 mm) (Biotic Chemical, New Taipei City, Taiwan) , 22% MeOH as the solvent, a flow rate of 3 mL·min^−1^, detection at 280 nm, and retention times (*Rt*) of 29, 31, and 38 min to obtain compounds **11** (4 mg), **10** (25 mg), and **13** (11 mg), respectively. PK-1-3-7 (0.25 g) was resubjected to Sephadex LH-20 column chromatography and developed with 95% ethanol to yield six fractions, PK-1-3-7-1 to PK-1-3-7-6. PK-1-3-7-3 was further purified using semipreparative HPLC with a Biosil 5 ODS-W column (10 × 250 mm), 17% acetonitrile as the solvent, a flow rate of 3 mL·min^−1^, detection at 280 nm, and *Rt* of 30 and 31 min to obtain compounds **3** (48 mg) and **4** (35 mg), respectively. PK-1-5-4 (3.90 g) was further isolated using a C_18_ column by a gradient elution of 100% water to 100% methanol; 8 fractions, PK-1-5-4-1 to PK-1-5-4-8, were obtained. PK-1-5-4-2 (0.48 g) was further eluted using a C_18_ column by a gradient elution of 100% water to 100% methanol; seven fractions, PK-1-5-4-2-1 to PK-1-5-4-2-7, were obtained. Compound **8** (238 mg) was obtained from the precipitate of PK-1-5-4-2-2. The filtrate of PK-1-5-4-1-1 was isolated by RP-HPLC (column: Biosil 5 ODS-W 10 mm × I.D. 250 mm; mobile phase: 55% methanol; flow rate: 3 mL·min^−1^; detector: RI (Bischoff Chromatography, Leonberg, Germany). Compounds **9** (6 mg) and **12** (6 mg) appeared at *Rt* of 12.0 and 12.5 min, respectively.

The *n*-BuOH extract (9.68 g) was also isolated using a Diaion HP-20 column (Sigma–Aldrich, St. Louis, MO, USA) by a gradient elution of 100% water to 100% methanol. The resulting eluates were assayed on a TLC plate, and 7 fractions (PKBu-1-1 to PKBu-1-7) were obtained. The fraction PKBu-1-2 was further isolated using a Sephadex LH-20 column by 95% ethanol to obtained compound **5** (56 mg). PKBu-1-3 (1.99 g) was further isolated using a C_18_ column by a gradient elution of 100% water to 100% methanol, and PKBu-1-3-1 to PKBu-1-3-6 were obtained. PKBu 1-3-3 (0.35 g) was further purified using semipreparative HPLC with a Biosil 5 ODS-W column (10 × 250 mm), 15% MeOH as the solvent, a 3 mL·min^−1^ flow rate, detection at 280 nm, and *Rt* of 18 and 20 min to obtain compounds **7** (10 mg) and **6** (14 mg). PKBu-1-4 was further isolated using a Sephadex LH-20 column (Pharmacia Biotech, Piscataway, NJ, USA) by 95% ethanol to obtain compound **2** (224 mg). The spectroscopic data obtained for compounds **1**–**8** and **10**–**12** were virtually identical to those reported in the literature. The identification data of two new compounds, **9** and **13**, are as follows:

#### 3.2.1. Compound **9**

An amorphous brown powder; UV (MeOH) λ_max_ (log ε): 316 (3.78), 290 (3.91), 263 (3.91) nm; electrospray ionization mass spectroscopy (ESI-MS) (Altrincham, Cheshire, UK) (positive) *m/z* 261.5 [M + H]^+^; HREIMS *m*/*z* 260.0687 (calculated for 260.0679); ^1^H-NMR (500 MHz, CD_3_OD) δ_H_ 7.21 (1H, s, H-1), 6.82 (1H, dd, *J* = 7.7, 1.0 Hz, H-7), 7.08 (1H, t, *J* = 7.7 Hz, H-8), 7.33 (1H, dd, *J* = 7.7, 1.0 Hz, H-9), 3.94 (3H, s, 2-OCH_3_), 4.15 (3H, s, 4-OCH_3_); ^13^C-NMR (125 MHz, CD_3_OD) (Table 1).

**Table 1:** NMR spectral data of 3,6-dihydroxy-2,4-dimethoxy-dibenzofuran (**9**) (δ values, in CD_3_OD, *J* in Hz).

Position	δc	δ_H_ (Mult, *J* in Hz)	HMBC
1	98.3	7.21 (1H, s)	C-9a, C-3, C-4a, C-2
2	147.4	-	-
3	140.2	-	-
4	134.7	-	-
4a	144.6	-	-
5a	146.0	-	-
6	144.1	-	-
7	113.5	6.82 (1H, dd, *J* = 7.7, 1.0)	C-5a, C-8
8	111.5	7.08 (1H, t, *J* = 7.7)	C-6, C-9a
9	124.5	7.33 (1H, dd, *J* = 7.7, 1.0)	C-7, C-9a
9a	127.9	-	-
9b	117.4	-	-
2-OMe	57.3	3.94 (3H, s)	C-2
4-OMe	61.3	4.15 (3H, s)	C-4

#### 3.2.2. Compound **13**

An amorphous brown powder; $[\alpha {]}_{\text{D}}^{24.5}$−10.6° (*c* 0.5, MeOH); UV (MeOH) λ_max_ (log ε): 265 (3.74) nm; ESI-MS (negative) *m/z* 393.1 [M − H]^−^; HRESIMS *m/z* 393.1202 [M − H]^−^ (calculated for 394.1264); ^1^H-NMR (500 MHz, CD_3_OD) δ_H_ 6.67 (1H, d, *J* = 1.8 Hz, H-2), 6.80 (1H, d, *J* = 1.8 Hz, H-6), 7.22 (1H, *J* = 7.6, 1.8 Hz, H-3′), 7.23 (1H, m, H-4′), 7.02 (1H, m, H-5′), 7.27 (1H, dd, *J* = 8.4, 1.4 Hz, H-6′), 5.03 (1H, d, *J* = 7.2 Hz, H-1′′), 3.43 (1H, m, H-2′′), 3.42 (1H, m, H-3′′), 3,34 (1H, m, H-4′′), 3.44 (1H, m, H-5′′), 3.68 (1H, dd, *J* = 12.0, 5.4 Hz, H-6′′), 3.86 (1H, dd, *J* = 12.0, 2.1 Hz, H-6′′), 3.86 (3H, s, 3′OCH_3_); ^13^C-NMR (125 MHz, CD_3_OD) (Table 2).

**Table 2:** NMR spectral data of 3,4-dihydroxy-5′-methoxybiphenyl-2′-*O*-β-d-glucopyranoside (**13**) (δ values, in CD_3_OD, *J* in Hz).

Position	δc	δ_H_ (Mult, *J* in Hz)	HMBC
1	130.6	-	-
2	111.4	6.67 (1H, d, *J* = 1.8)	C-3, C-4, C-6, C-1′
3	146.0	-	-
4	134.6	-	-
5	149.1	-	-
6	106.8	6.80 (1H, d, *J* = 1.8)	C-2, C-4, C-5, C-1′
1′	133.0	-	-
2′	155.4	-	-
3′	116.4	7.22 (1H, dd, *J* = 7.6, 1.8)	C-1′, C-2′, C-5′
4′	129.0	7.23 (1H, m)	C-2′, C-5′
5′	123.4	7.02 (1H, m)	C-1′, C-3′, C-4′, C-6′
6′	131.7	7.27 (1H, dd, *J* = 8.4, 1.4)	C-1, C-2′, C-4′
1′′	101.8	5.03 (1H, d, *J* = 7.2)	C-2′
2′′	75.0	3.43 (1H, m)	C-1′′
3′′	78.2	3.42 (1H, m)	C-4′′
4′′	71.3	3.34 (1H, m)	C-4′′
5′′	78.3	3.44 (1H, m)	C-4′′, C-3′′
6′′	62.5	3.68 (1H, dd, *J* = 12.0, 5.4); 3.86 (1H, dd, *J* = 12.0, 2.1)	C-5′′
5-OCH_3_	56.8	3.86 (3H, s)	C-5

### 3.3. Cell Culture

Normal HEMn cells (C-102-5C, Cascade Biologics, Inc., Portland, OR, USA) obtained from neonatal foreskin were grown in Medium 254, which contains essential and nonessential amino acids, vitamins, other organic compounds, trace minerals, and inorganic salts (Cat. No. M-254-500; Gibco, Portland, OR, USA), supplemented with human melanocyte growth supplement (Cat. No. S-002-5; Gibco).

### 3.4. Cell Viability in Human Epidermal Melanocytes

The cell viability of HEMn was determined using the WST-8 method. In brief, cells were plated at 10^5^/well (in 24-well plates). After 24 h of culture, the test samples were treated and incubated for an additional 24 h. The optical density was measured at 450 nm on a µQuant microplate reader (Bio-Tek Instruments, Vermont, VT, USA). The viability of the melanocytes was calculated using the following formula: (absorbance of sample tested/absorbance of medium only) × 100%.

### 3.5. Tyrosinase Activity in Human Epidermal Melanocytes

Cellular tyrosinase activity was measured using a previously described method [51]. In brief, HEMn cells were cultured in the wells of a 24-well plate. After treatment with the tested compounds for 24 h, the cells were washed with potassium phosphate-buffered saline (PBS) and lysed with PBS (pH 6.8) containing 1% Triton X-100. The cells were disrupted by freezing and thawing, and the lysates were clarified by centrifugation at 10,000× *g* for 10 min. The protein content was determined using a BCA Protein Assay Kit (Pierce Biotechnology, Inc., Rockford, IL, USA). Each well of a 96-well plate contained 40 μg of protein, 2.0 mM l-DOPA, and 0.1 M PBS (pH 6.8). After incubation at 37 °C for 1 h, the absorbance was measured at 475 nm by using an enzyme-linked immunosorbent assay reader. Tyrosinase activity was calculated using the following formula: Tyrosinase activity (%) = (OD_475_ of sample/OD_475_ of control) × 100%.

### 3.6. Melanin Contents in Human Epidermal Melanocytes

Melanin contents were measured as described previously [51]. Briefly, HEMn cells were treated with tested samples for 24 h. Cell pellets were lysed with 1 N NaOH at 37 °C overnight and centrifuged for 10 min at 10,000× *g*. Relative melanin content was measured at 450 nm using an ELISA reader (Bio-Tek Instruments).

### 3.7. Western Blot Analysis

Western blot analysis was performed as described previously [52]. The cells (1 × 10^6^) were collected and lysed with iced PBS containing 1% Triton X-100, 1 mM phenylmethylsulfonyl fluoride, 1 μg·mL^−1^ aprotinin, and 10 μg·mL^−1^ leupeptin. The cell lysates were subjected to centrifugation at 12,000× *g* for 5 min, and the supernatant protein was quantified using a BCA Protein Assay Kit (Pierce Biotechnology, Inc.). Samples (with equal amounts of protein) were added to equal volumes of a sodium dodecyl sulfate (SDS) sample buffer and boiled for 5 min prior to separation by 10% SDS–polyacrylamide gel electrophoresis. They were then electrotransfered to polyvinylidene fluoride (PVDF) membranes (Immobilon-P; Millipore Corp., Bedford, MA, USA). The membranes were incubated overnight with a blocking solution containing 5% skim milk. Anti-TYR (C-19), anti-TRP1 (G-17), and anti-TRP2 (D-18) (Santa Cruz Biotechnology, Inc., California, CA, USA) antibodies served as the primary antibodies at 1:1000 dilution and were incubated with the PVDF membranes at room temperature for 2 h. After extensive washes, the blots were incubated with alkaline phosphatase (AP)-conjugated antigoat IgG (Santa Cruz Biotechnology) at 1:5000 dilution for 1 h at room temperature. The AP activity was detected using the nitro blue tetrazolium/5-bromo-4-chloro-3-indolyl phosphate substrate. β-actin was used as the internal control. The relative intensities of each band were calculated for each intensity value (intensity × area) by using the Quantity One 1-D Analysis Software (Bio-Rad, New York, NY, USA); the values were normalized to the intensity values of the control.

### 3.8. Real-Time PCR Analysis

Quantification of genes transcript by real-time PCR was performed using a LightCycler^®^ 480 TaqMan (Roche, Mannheim, Germany) according to the manufacturer’s instructions. The mRNA was extracted with high pure RNA isolation kit (Roche) and the quality of the total RNA was evaluated using Nano Drop. Relative ratio of a target gene expression was calculated with the CP value by the LightCycler4 Data analysis software automatically. MITF forward primer: CAAAAGTCAACCGCTGAAGA, reverse primer: AGGAGCTTATCGGAGGCTTG; PAX3 forward primer: TTGGCAATGGCCTCTCAC, reverse primer AGGGGAGAGCGCGTAATC; GAPDH forward primer: AGCCACATCGCTCAGACAC, reverse primer: GCCCAATACGACCAAATCC.

### 3.9. Kinetic Analysis of Cellular Tyrosinase

To examine the kinetic mechanism, the tested compounds were analyzed using a cellular tyrosinase assay. HEMn cells were cultured in the wells of a 24-well plate. After treatment with the tested compounds for 24 h, the cells (1 × 10^5^) were collected and lysed with iced PBS containing 1% Triton X-100. After being disrupted by freezing and thawing, the lysates were clarified by centrifugation at 10,000× *g* for 10 min. Each well of a 96-well plate contained 40 μg of protein, various concentrations of the l-DOPA substrate (62.5, 125, 250, 500, 1000, and 2000 µM), and a phosphate buffer (pH 7.4), then incubated at 37°C for 1 h. The absorbance was measured at 475 nm by using a μQuant microplate reader (Bio-tek Instruments). The apparent inhibition constants for the isolated compounds were calculated using Lineweaver-Burk plots.

### 3.10. Statistical Analysis

Differences in data were evaluated for statistical significance (*p* < 0.05) by using the Student’s *t*-test.

## 4. Conclusions

We evaluated the tyrosinase-inhibitory effects of *P. koidzumii*, which is a plant native to Taiwan, in HEMn cells. In total, 13 compounds were identified in the present study, including two new compounds, compounds **9** and **13**, as well as 11 known compounds. The new 3,4-dihydroxy-5-methoxybiphenyl-2′-*O*-β-d-glucopyranoside (**13**) exhibited inhibitory effects on the protein expression of tyrosinase and TRP2, as well as the mRNA expression of PAX3 and MITF in a concentration-dependent manner. It also acted as an uncompetitive mixed-type inhibitor in kinetic studies. The results indicate that the active compound **13** from *P. koidzumii* may be a potential candidate as a tyrosinase inhibitor for application in skin care cosmetics.
